# Empathy in AI for Health and Care Settings—Definition, Expression, and Measurement: Protocol for a Scoping Review

**DOI:** 10.2196/93078

**Published:** 2026-05-05

**Authors:** Alastair Howcroft, Steve Benford, Michel Valstar, Holly Blake

**Affiliations:** 1 School of Computer Science University of Nottingham Nottingham, England United Kingdom; 2 BLUESKEYE AI Ltd Nottingham, England United Kingdom; 3 Queen’s Medical Centre School of Health Sciences University of Nottingham Nottingham, England United Kingdom; 4 NIHR Nottingham Biomedical Research Centre Nottingham University Hospitals NHS Trust Nottingham, England United Kingdom

**Keywords:** empathy, artificial intelligence, AI, affective computing, chatbots, conversational agents, social robots, natural language processing, human-computer interaction, health care, patient-clinician relations, patient care, scoping review

## Abstract

**Background:**

“Empathy” is widely discussed in health and care settings and is increasingly claimed as an attribute of artificial intelligence (AI) systems (eg, socially assistive robots and chatbots), but the term is used inconsistently across the literature. In research on AI in these settings, it is often unclear what authors mean by “empathic AI,” what systems do that is intended to be empathic, and how empathy is assessed. This matters because perceived empathy can shape users’ experience of AI-mediated support and their willingness to engage with these systems.

**Objective:**

This study aims to map how empathy is defined, operationalized, and evaluated in peer-reviewed AI research in health and care settings and to describe interactional design features commonly reported in systems perceived as more empathic.

**Methods:**

This protocol outlines a scoping review following Joanna Briggs Institute guidance and is reported in accordance with PRISMA-ScR (Preferred Reporting Items for Systematic Reviews and Meta-Analyses Extension for Scoping Reviews). We use “AI” as an umbrella term and will extract and classify each system’s type (eg, rule-based or large language model–based). We will search PubMed (MEDLINE), Embase, PsycInfo, CINAHL, Scopus, IEEE Xplore, and the ACM Digital Library databases. Two reviewers will screen titles and abstracts using ASReview and full texts by using Rayyan. We will extract study characteristics, empathy definitions and framing, empathy-related system behaviors and design features, and evaluation methods, and synthesize findings thematically.

**Results:**

This scoping review forms a part of the first author's doctoral research, funded by an Engineering and Physical Sciences Research Council studentship from October 2025. Pilot searches were conducted on January 20, 2026; full searches and synthesis are planned for 2026, with publication anticipated in 2027. The review will produce (1) a summary of how empathy is defined in AI research in health and care settings, (2) a grouped list of the main empathic interactional behaviors and design features described, and (3) an overview of how empathy is measured across studies. Where studies report empathy ratings, we will summarize which features are most commonly present in higher-rated systems within comparable contexts.

**Conclusions:**

The review will provide a clearer picture of what researchers mean by “AI empathy” in health and care settings and what system features are most commonly used when trying to build it. These findings may help guide the development of more empathic AI systems.

**International Registered Report Identifier (IRRID):**

PRR1-10.2196/93078

## Introduction

When we describe someone as empathetic (or not), most people have a general sense of what we mean. However, things become murkier when we try to define what “empathy” actually is. There are many different definitions of empathy. This is partly because the term is used in different contexts, such as health care [1], psychology [2], philosophy [3], neuroscience [4], and even machine learning [5]. This lack of consistency can quickly complicate attempts to reason clearly about empathy, particularly in the context of artificial intelligence (AI). In fact, as early as 1935, psychoanalyst Theodor Reik was warning that “the concept of empathy...has come to mean so much that it is beginning to mean nothing” [6].

When we ask Google to define empathy, it returns a definition from Oxford Languages: “the ability to understand and share the feelings of another” [7]. This is generally how the word is understood today. However, the inclusion of the term *share* raises an important ambiguity: if someone feels sad, do I understand that they are sad, or do I feel a bit sad knowing they are sad—and which one is “empathy”? This distinction is particularly important for discussions of AI and empathy since AI cannot literally feel emotions like humans. Because the term “AI” is used inconsistently across communities, we use it here to refer to interactive computational systems that generate user-facing outputs. In AI, systems designed to respond empathically are often described in terms of *artificial* or *computational* empathy [8].

Empathy comes from the German word “Einfühlung,” coined by philosopher Robert Vischer in 1873, and literally meaning “feeling-into” [9]. Originally, it referred to an esthetic process of appreciating art by projecting one’s own subjective experience into an artwork or other object of beauty. For example, by imagining oneself in a painted landscape, one might feel the isolation of a desolate scene, or by imagining oneself as a towering sculpture, one might feel height or dominance. By 1909, the concept had entered English as “empathy” [10], though it largely retained this esthetic “feeling-into” sense, rather than the understanding of other minds [11]. Only after World War II in the late 1940s did empathy acquire its modern interpersonal sense, as it was taken up in social psychology [11,12], akin to the idea of “putting yourself in someone else’s shoes.”

While some definitions of empathy have included the idea of sharing or “entering into” another person’s feelings since at least the early 1950s [13], a distinct interpretation emerged within health care during the 1950s and 1960s [1]. In health care contexts, empathy increasingly came to be understood as understanding another person’s feelings rather than experiencing those feelings oneself. Nowadays, outside health care, empathy is often taken to involve both understanding and sharing another person’s emotions; by contrast, in health care settings it is typically defined as a practitioner’s ability to understand another person’s emotional experience—without sharing the same feelings—and to communicate that understanding [14-16]. This health care–oriented construct is often labeled *therapeutic empathy* and is applied across health care settings, such as nursing [17] and counseling [14].

We distinguish empathy from the related constructs of sympathy and compassion, consistent with how patients often describe these terms [18]. In patients’ accounts, sympathy is an emotionally distant, pity-based response that lacks genuine understanding, whereas empathy is an emotionally attuned understanding of the person. Compassion goes further by coupling this empathic understanding with an action-oriented, altruistic intent to alleviate suffering. To clarify the terminology used in this review, Table 1 summarizes the key constructs and distinctions relevant to empathy in AI and health care.

**Table 1:** Key empathy-related constructs and distinctions relevant to this review.

Term	Conceptual framing	Key distinction	Components identified in the literature
Empathy (general or modern use)	Commonly understood as the ability to understand and share another person’s feelings	Involves both understanding another person’s emotions and vicariously feeling some version of them oneself	According to a review of reviews [19], understanding the other person’s experience (recognizing what they think or feel); an affective response appropriate to their situation; sharing their feelings; and maintaining self-other differentiation (staying aware that the feeling belongs to the other person, not to you)
Therapeutic empathy (empathy in health care)	Understanding another person’s emotional experience—without sharing their feelings—and communicating that understanding	Differs from everyday use because it focuses on cognitive understanding (rather than affective sharing) and communication	According to a thematic analysis of existing definitions [16], discovering the patient’s experience (eg, by listening); understanding it (using imagination and reasoning); checking that understanding with the patient (by communicating their understanding back and checking its accuracy); showing some emotional response (such as concern, rather than feeling the same emotion); engaging in caring behaviors to help the patient; and maintaining boundaries
Artificial empathy	The outward expression of empathy by an AI system, manifested through behaviors intended to reflect empathic understanding	Concerned with the observable, interactional expression of empathy—what a system does—rather than how empathic behavior is computationally modeled or generated	This review will map which behaviors, cues, and communicative strategies are described as conveying empathic understanding in the health and care literature on AI and robotics (eg, nonverbal cues such as nodding [20] and linguistic strategies that acknowledge the user’s feelings [21])
Computational empathy	The technical processes by which an AI system models, detects, and generates empathic responses	Concerned with underlying implementation—how empathic behavior is modeled and generated—rather than its outward expression	A review of affective computing [22] identifies relevant computational processes, including methods for detecting or inferring users’ emotional states from visual, vocal, textual, or multimodal signals—though as this review focuses on artificial empathy, these underlying processes are not examined in depth
Sympathy (in health care; patient perspective)	In patients’ accounts, sympathy, while well-intentioned, is described as a pity-based response that lacks genuine understanding of the person’s experience	Unlike empathy, sympathy is not characterized by emotional attunement or an attempt to understand the person’s experience; rather, it is experienced as feeling sorry for them	In a grounded theory study of patients [18], sympathy was described as a pity-based response, marked by a lack of genuine understanding of the person’s experience, focused more on the observer’s own emotional comfort than on the patient’s needs, and generally experienced as shallow, obligatory, and unhelpful By contrast, empathy was understood as recognizing someone’s situation and trying to understand how they feel That said, expressions of sympathy can serve a meaningful role in care—for example, personalized sympathy cards have been valued by bereaved families [23]
Compassion (in health care; patient perspective)	Compassion goes further than empathy by coupling empathic understanding with an action-oriented, altruistic intent to alleviate suffering	Adds helping intention and action beyond empathic understanding	In the same grounded theory study of patients [18], compassion was described as including understanding the person’s experience, an altruistic orientation to their needs, action aimed at helping, and small acts of kindness that expressed care and concern

More recently, explicit references to empathy have increased sharply in the arXiv literature (a major preprint archive for AI and computer-science research), with the number of submissions per year containing *empathy*, *empathic*, or *empathetic* in the title or abstract rising from 2 in 2015 to 42 in 2020 and 328 in 2025 [24]. By contrast, equivalent searches for sympathy and compassion returned far smaller increases over the same period (from 20 to 38 and from 74 to 131, respectively, between 2015 and 2025). Much of the empathy-focused research concerns enabling, measuring, or evaluating empathetic behavior in AI systems—particularly large language model–based agents—and is often situated in health care, the leading end-user segment for affective computing [25].

As Hackney [14] remarked, “the meaning of a construct is determined by those who use it as much as by the setting in which it is used.” In the context of AI in health care, this means that *artificial empathy* must be understood not only through inherited theoretical definitions but also through how the term is actually used, specified, and enacted in the literature. Although prior work has synthesized definitions of therapeutic empathy in health care more broadly [16], no such review has mapped how empathy is both explicitly defined and operationalized in AI–health care systems in the literature. As AI becomes more embedded in health care, clarifying what researchers mean by “empathy”—and what they measure or build when they claim it—becomes increasingly important. This scoping review aims to address this gap by examining how empathy is defined in AI–health care papers. It will also investigate how conveying empathy is operationalized in practice, including empathy-related system behaviors and design features (eg, nonverbal cues such as nodding to convey understanding [20] and linguistic strategies that signal recognition and acknowledgment of the user’s feelings [21]), as well as the methods used to assess or measure perceived empathy (eg, participant ratings on Likert scales or validated questionnaires). This review is concerned with what we term *artificial empathy*—empathy as expressed and observable in interaction—rather than *computational empathy*, understood as the underlying processes used to detect emotion or generate responses (eg, automatic audio, visual, and physiological emotion analysis [26]). These processes are less visible to users and less relevant from a *purely* interactional standpoint. By examining how empathy is defined, operationalized, and evaluated, the review aims to clarify what “AI empathy” currently denotes in health care research and to establish a clearer account of what artificial empathy means in practice. Examining how empathy is operationalized will also help identify how AI systems are designed to appear empathic, and how this might be done more deliberately. Where reported, empathy evaluation outcomes will be mapped to described system behaviors and design features to identify recurring patterns associated with higher perceived empathy.

## Methods

### Guiding Frameworks

This review will be conducted in accordance with the Joanna Briggs Institute methodology for scoping reviews, which provides a clear approach for systematically identifying and mapping the literature. The review will be reported in line with the PRISMA-ScR (Preferred Reporting Items for Systematic Reviews and Meta-Analyses Extension for Scoping Reviews) guidance, which standardizes how scoping reviews describe their search and study selection process [27]. Together, these frameworks help ensure the review is structured, transparent, and not based on an ad hoc reading of the literature. “AI system” refers to the interactive application or agent studied (eg, a chatbot or social robot), including whatever underlying approach it uses to generate its outputs. “Health care” is used broadly to cover health and care contexts, including clinical, therapeutic, and caregiving settings. We use the terms empathic and empathetic interchangeably, reflecting common use in the literature.

### Objectives and Review Questions

The review aims to clarify what “empathy” currently denotes in AI–health care research by examining both explicit definitions in conjunction with their practical operationalization (eg, system capabilities and evaluation approaches). The review aims to address the following review questions (RQs):

- RQ1: How is empathy defined in AI–health care publications?
- RQ2: How is empathy outwardly operationalized in AI–health care systems (eg, interactional behaviors, communicative strategies, and design features claimed to be “empathetic”)?
- RQ3: How do studies measure empathy in terms of specific behaviors or design features?
- RQ4: When empathy is evaluated, what empathy-related system behaviors or design features are reported in systems with higher perceived empathy (within comparable measures and contexts)?

We focus on empathic behaviors and design features as they are expressed in interaction and assessed by users or researchers, rather than on internal processes such as emotion detection or response selection.

### Eligibility Criteria

Eligibility criteria were developed to identify studies focused specifically on artificial empathy in AI systems used in health and care settings. Inclusion and exclusion criteria are summarized in Textbox 1.

**Inclusion and exclusion criteria.**

**Inclusion criteria**

Studies must meet all 3 conditions:

- The primary context is a health or care setting (eg, counseling, clinical care, and caregiving).
- An AI system produces interactional outputs intended to communicate with or respond to a user (eg, dialogue, voice, avatar, or social robot).
- Empathy is a clear focus, meaning the study either explicitly defines or frames empathy in relation to how it is expressed in interaction or specifies empathy-related system behaviors, communicative strategies, or design features—even without a formal definition.

**Exclusion criteria**

- Purely theoretical or speculative studies with no link to what an AI system does or how it is assessed in a health or care context.
- Studies that do not produce interactional outputs.
- Non–peer-reviewed studies (eg, preprints).
- Studies that treat “empathy” only as an outcome label or simple judgment without defining it or specifying the system behaviors involved.
- Studies that define empathy solely in terms of computational processes (eg, emotion detection) with no account of how these shape user-facing behavior.
- Studies focused on general prosocial behavior (eg, sociability, warmth, or friendliness) rather than empathy specifically.

This review focuses on what a system does that is intended to be empathic in interaction (artificial empathy), not the underlying computational processes used to generate responses (computational empathy). Computational processes such as sentiment analysis are not sufficient on their own; a paper must describe how they shape the system's user-facing behavior.

**Figure 1:**
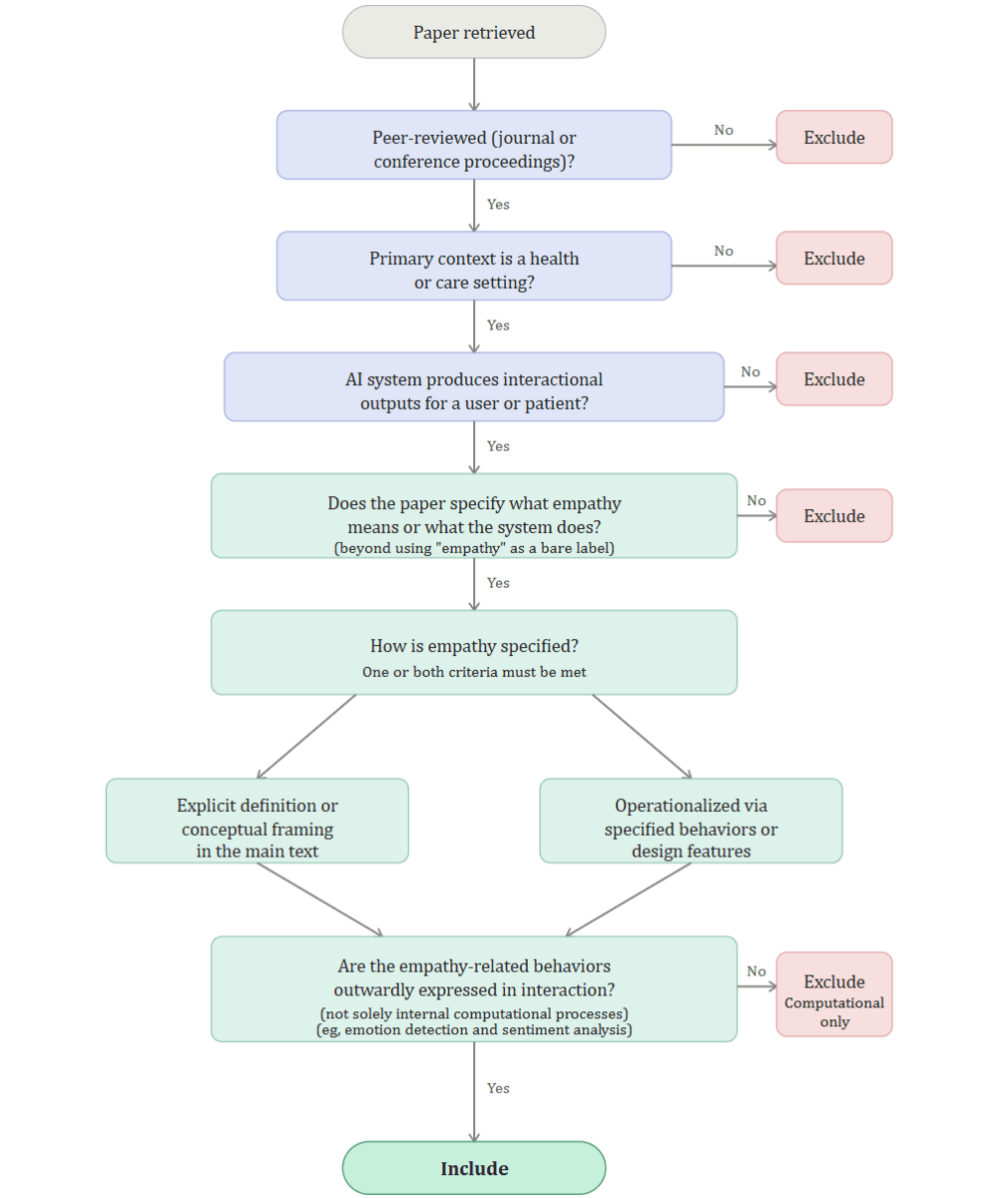
Study selection decision flowchart showing the eligibility criteria used to determine inclusion and exclusion. AI: artificial intelligence.

Studies that treat empathy only as an outcome label or simple judgment without defining it or specifying empathy-specific design features are excluded; for example, some studies rate a large language model’s output for “empathy” without describing any empathy-specific design features (eg, [28]). The goal is to analyze studies where empathy is specified meaningfully—defined or operationalized through described system behaviors. Figure 1 illustrates how these eligibility criteria are applied during study selection.

### Information Sources and Search Strategy

We will search PubMed (MEDLINE), Embase, PsycInfo, CINAHL, Scopus, IEEE Xplore, and the ACM Digital Library databases from inception to May 31, 2026 (anticipated date). Reference lists of included studies and relevant reviews will be screened.

The search strategy is designed to identify peer-reviewed studies on empathy in AI systems used in health care. It will focus on 3 key groups of terms: empathy, AI technologies, and health care context. Broad terms for AI and health care will be used to capture diverse technologies and settings, while empathy terms (“empathy,” “empathic,” “empathetic,” and derivatives) will be narrower to maintain specificity. While terms such as warmth, rapport, or sympathy can sometimes overlap with empathy, they are not equivalent to it. Empathy is a distinct construct, generally understood to involve recognizing and responding to another person’s feelings—whereas warmth may simply mean sounding pleasant, and sympathy may involve expressing sorrow without engaging with the person’s specific emotional experience. Broadening the search to encompass all prosocial behaviors would dilute the very specificity the review aims to provide. This review is specifically concerned with empathy, so studies using only these adjacent terms without framing their focus as empathy fall outside its scope. Term groups will be combined with “OR” within categories and “AND” across categories. No date limits will be applied.

We developed a preliminary PubMed database search and provided the full PubMed strategy in Multimedia Appendix 1. This PubMed strategy will be adapted for each additional database. The full search strategy for each database will be provided in a multimedia appendix in the published review.

### Study Selection

Title and abstract screening will be conducted in ASReview (open-source active-learning software) [29]. Two reviewers will screen titles and abstracts independently in separate ASReview projects; ASReview will be used as the screening platform and to prioritize the order in which records are presented, but all retrieved records will be screened. Screening decisions will be exported and cross-referenced to identify agreements and disagreements, with conflicts resolved through discussion (and adjudication by an additional reviewer if required).

Full-text screening will then be conducted in Rayyan [30], with 2 reviewers independently assessing all eligible full texts against the inclusion criteria. Disagreements at full text will be resolved through discussion, with adjudication by an additional reviewer if required.

### Data Collection

The primary reviewer (AH) will develop the data extraction form and extract all data from all included studies, including detailed technical information about the AI systems and conceptual data on empathy (definitions and framing, system behaviors, and evaluation approaches). A secondary reviewer will independently extract key nontechnical study characteristics (eg, bibliographic details, study design and health care setting, country, participant or user group, and interaction modality) and will verify a subset of AH’s extracted technical and empathy-related data (approximately 10%-20% of included studies).

From all included studies, we will extract bibliographic details (year and venue), study design and health care setting, country, participant or user group, AI system type and interaction modality, any explicit empathy definition or cited framing in relation to how empathy is expressed in interaction, how empathy is outwardly operationalized in the system, and how empathy is evaluated or measured, including global empathy ratings where used, as well as scales or annotation procedures tied to specific behaviors or features where available.

Where studies report empathy evaluation findings and describe specific system behaviors or features, we will relate these to identify patterns associated with higher perceived empathy where possible. Because studies using only a global empathy rating without defining empathy or specifying system behaviors are excluded (refer to the exclusion criteria in Textbox 1), the review’s account of evaluation methods reflects only studies that meet a minimum threshold of conceptual engagement with empathy. Table 2 summarizes the data extraction framework.

**Table 2:** Data extraction framework showing how each column maps to the review questions (RQ)^a^.

Component	Description	Illustrative example	Review question addressed
Definition	How empathy is defined or framed in relation to how it is expressed in interaction	Empathy defined as understanding a patient’s feelings and communicating that understanding	RQ1
Features	Interactional behaviors, communicative strategies, and design features intended to convey empathy	Robot head nodding, acknowledging feelings, prosodic changes, and use of a user’s name	RQ2
Evaluation methods	Methods or tools used to assess empathy (eg, scales, coding schemes, or qualitative approaches)	Perceived empathy rated on a Likert scale; participants annotated empathic behaviors	RQ3
Empathy findings	Evaluation findings reported by the studies	Empathetic condition rated significantly more empathic by users (*P*<.001)	RQ4

^a^Inclusion requires that a study populates the Definition row, the Features row, or both. A study may contribute data to 1, 2, 3, or all 4 components (eg, a study might only define empathy without describing specific features or evaluating them).

### Synthesis Plan

A narrative synthesis will be used to collate, summarize, and report findings. We will first produce descriptive summaries (eg, counts and tabulations) of study characteristics, including health care setting, user group, AI system type, interaction modalities, and empathy evaluation approaches. We will then conduct a thematic analysis of how empathy is defined, what AI systems do that is described as empathic, and how empathy is measured or labeled. Finally, we will summarize the most common empathy-related design features and compare how definitions, behaviors, and measurement approaches align or differ. The synthesis will produce a map of definitions, a taxonomy of empathic behaviors (eg, linguistic strategies and other modalities), a comparison of measurement strategies, and a set of gaps and practical pointers to support more rigorous design and evaluation of empathic AI in health care. In addition, we will construct a feature-outcome map linking the empathy-related behaviors and design features described in each study to the reported empathy evaluation findings (quantitative and qualitative) to identify recurring patterns. Because studies rarely evaluate empathy at the level of individual features, we will not attribute empathy scores to specific components or make causal claims; rather, we will describe patterns within comparable measures and contexts.

### Quality Appraisal

No formal quality appraisal will be undertaken, as the aim of this scoping review is to map how empathy is defined, operationalized, and evaluated in AI–health care research rather than to estimate intervention effects.

### Ethical Considerations

Ethics approval is not required for this review, as it synthesizes published literature.

### Dissemination Plan

The findings of this scoping review will be disseminated through peer-reviewed, open-access publication and, where appropriate, presentations at relevant conferences in fields such as human-robot interaction, affective computing, human-computer interaction, and digital health (eg, ACM/IEEE International Conference on Human-Robot Interaction, International Conference on Affective Computing and Intelligent Interaction, ACM Conference on Conversational User Interfaces, ACM Conference on Human Factors in Computing Systems). We will produce a plain-language summary for nonacademic audiences, including patient and public involvement partners, designers and developers of AI and robotic systems, and health and care organizations interested in the development or evaluation of AI-enabled tools. Key insights relevant to the design, evaluation, and governance of empathic AI in health and care will be shared with professional bodies and stakeholder networks across both the computing and health care communities. Supplementary materials, including the full search strategies and data extraction framework, will be made available alongside the published review.

### Protocol Amendments

Any deviations from this protocol (eg, changes to eligibility criteria, information sources, or analysis approach) will be documented and reported in the final manuscript.

## Results

**Figure 2:**
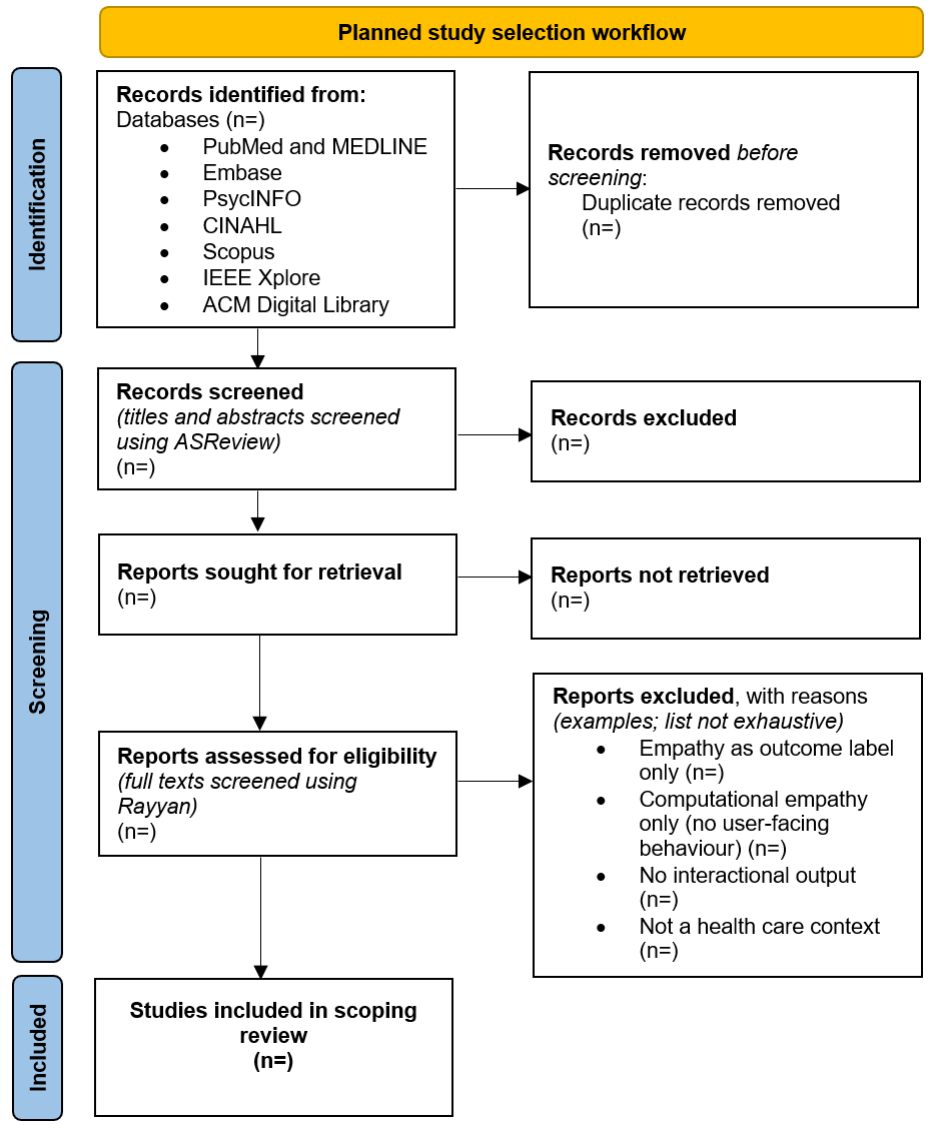
Planned study selection workflow to be reported using the PRISMA (Preferred Reporting Items for Systematic Reviews and Meta-Analyses) 2020 flow diagram. Example exclusion reasons are shown; counts will be added once screening is complete.

This scoping review is being conducted as part of the first author's (AH) doctoral research on AI and Feelings in Computer Science at the University of Nottingham, supported by an Engineering and Physical Sciences Research Council (EPSRC) studentship within the Somabotics project (from October 2025). The study selection procedure will be illustrated using the PRISMA (Preferred Reporting Items for Systematic Reviews and Meta-Analyses) 2020 flow diagram [31], following the planned workflow shown in Figure 2. Pilot searches were conducted on January 20, 2026, to assess the initial relevance of the results (Multimedia Appendix 1). Full database searches are planned for May 2026, with screening, data extraction, and synthesis to follow through late 2026. The completed review is expected to be submitted in late 2026 or early 2027, with publication anticipated in 2027. The review results will address what definitions of “empathy” are used in AI in health care publications, what AI systems actually do that is intended to be empathic, how researchers measure or judge whether an AI is empathic, and which observable AI features are most often linked to higher empathy ratings (where possible).

## Discussion

### Significance and Implications

AI systems such as triage chatbots and social or companion robots (eg, in older adult care) are increasingly used in health care, making empathy an important design and evaluation consideration for creating more supportive human-AI interactions [8,21]. Empathic care is associated with better outcomes, including greater trust and engagement [32], whereas its absence is associated with frustration, disappointment, avoidance of care, and—in some therapeutic contexts—worse treatment outcomes [33,34]. Evidence suggests that, at least in text-based interactions, AI systems can be perceived as more empathic than human health care practitioners [35]. Although some argue that AI cannot be empathic because it cannot feel [36], this applies a stronger standard than is typically used in health care, where empathy is usually defined in cognitive terms—understanding a patient’s feelings and communicating that understanding [14-16]. Under this health care–oriented definition, AI systems can plausibly meet empathic criteria. Therefore, this review aims to clarify what “empathy” means in AI–health care research, how it is operationalized in system design, and which features are most commonly associated with higher perceived empathy. While the review contributes theoretically, it is intended to be practical, informing how AI systems might be designed to feel empathic to users, thereby improving the quality of AI-mediated care and supporting better outcomes for service users. The review may also identify gaps in operationalization, including limited attention to certain interaction modalities (eg, haptic and tactile feedback) relative to text and audiovisual approaches, suggesting directions for future work. At the same time, findings should be interpreted cautiously given ethical concerns (eg, overreliance or dependence on empathic AI) [8], concerns that technology should not entirely replace humans in the care process [37], and the possibility that overtly “humanlike” empathic behavior may feel uncanny or insincere [21].

### Strengths and Limitations

This review has several strengths. It is methodologically rigorous, using a transparent, systematic approach informed by established scoping review guidance (Joanna Briggs Institute and PRISMA-ScR) and drawing on interdisciplinary sources to support a comprehensive mapping of the field. It is also conceptually disciplined: rather than asking “does this paper mention empathy?”, it requires studies to either define empathy or describe empathy-specific design features, giving the review substantive analytic value in mapping what researchers actually mean by “empathic AI.” These features position the review to produce findings that are both broad in scope and analytically meaningful.

This focus comes with trade-offs. First, by excluding studies that treat empathy only as a label, our account reflects only studies meeting a minimum threshold of conceptual engagement, prioritizing analytic clarity over breadth; this may exclude a substantial portion of the literature in which empathy is used more loosely. Second, because the review is focused specifically on empathy, it will not capture communication competence in AI more broadly [38] or adjacent constructs such as warmth, rapport, or supportive communication, even though these may overlap with or contribute to users’ perceptions of empathic interaction. Third, by restricting the review to peer-reviewed literature, we may miss recent developments in this fast-moving area (eg, on arXiv), and emerging or proprietary AI systems may not be fully represented in the published literature. Fourth, because studies typically bundle multiple empathy-related features into a single condition, we can identify patterns linking features to higher empathy ratings but cannot attribute outcomes to individual features. Finally, by focusing on outward expressions of empathy, we do not examine the computational processes used to detect emotion or generate empathic responses [22,26], which can be more typical concerns of affective computing research.

## Data Availability

No new datasets were generated or analyzed for this scoping review protocol; all data will be derived from publicly available published studies.

## Appendix

Multimedia Appendix 1 A preliminary PubMed database search strategy.

## Abbreviations

AI: artificial intelligence
PRISMA: Preferred Reporting Items for Systematic Reviews and Meta-Analyses
PRISMA-ScR: Preferred Reporting Items for Systematic Reviews and Meta-Analyses Extension for Scoping Reviews
RQ: review question
