# Supplementary material for: Empathy in AI for Health and Care Settings—Definition, Expression, and Measurement: Protocol for a Scoping Review
Source: JMIR Res Protoc. 2026 May 5;15:e93078. doi: 10.2196/93078 (PMC13187702; doi:10.2196/93078)
Supplement: Multimedia Appendix 1 [file resprot_v15i1e93078_app1.docx]

# Multimedia Appendix A: Search strategy

PubMed
Search conducted on January 20, 2026

| **Search** | **Query** | **Records retrieved** |
| --- | --- | --- |
| #1 | ("Empathy"[Mesh] OR empath*[tiab]) | 44,700 |
| #2 | ("Artificial Intelligence"[Mesh] OR "Machine Learning"[Mesh] OR "Natural Language Processing"[Mesh] OR "Neural Networks, Computer"[Mesh] OR "Robotics"[Mesh] OR "Artificial Intelligence"[tiab] OR AI[tiab] OR "machine learning"[tiab] OR "deep learning"[tiab] OR "large language model"[tiab] OR "large language models"[tiab] OR LLM[tiab] OR LLMs[tiab] OR "generative AI"[tiab] OR chatbot*[tiab] OR "conversational agent*"[tiab] OR "dialogue system*"[tiab] OR "virtual assistant*"[tiab] OR "voice assistant*"[tiab] OR "intelligent agent*"[tiab] OR "social robot*"[tiab] OR avatar*[tiab] OR "embodied conversational agent*"[tiab]) | 522,343 |
| #3 | ("Delivery of Health Care"[Mesh] OR "Health Care Sector"[Mesh] OR "Patient Care"[Mesh] OR "Health Services"[Mesh] OR "Hospitals"[Mesh] OR "Primary Health Care"[Mesh] OR "Mental Health Services"[Mesh] OR "Counseling"[Mesh] OR "Psychotherapy"[Mesh] OR "Nursing"[Mesh] OR healthcare[tiab] OR "health care"[tiab] OR clinical[tiab] OR patient*[tiab] OR clinic*[tiab] OR hospital*[tiab] OR medicine[tiab] OR nursing[tiab] OR counseling[tiab] OR counselling[tiab] OR therapy[tiab] OR "mental health"[tiab] OR caregiv*[tiab] OR "long term care"[tiab] OR "home care"[tiab]) | 15,716,715 |
| #4 | 1 AND 2 AND 3 | 768 |
